# Angiomatous, Predominantly Cystic Meningiomas: A Case Report and Literature Review of a Unique Group of Meningiomas

**DOI:** 10.7759/cureus.85513

**Published:** 2025-06-07

**Authors:** Carmine Romano, Jens Schittenhelm, Marcos Tatagiba, Felix Behling

**Affiliations:** 1 Department of Neurosurgery, San Carlo Regional Hospital, Potenza, ITA; 2 Department of Neuropathology, University Hospital Tübingen, Eberhard Karls University Tübingen, Tubingen, DEU; 3 Department of Neurosurgery and Neurotechnology, University Hospital Tübingen, Eberhard Karls University Tübingen, Tübingen, DEU; 4 Department of Neurosurgery and Neurotechnology, University Hospital Tübingen, Eberhard Karls University Tübingen, Tubingen, DEU

**Keywords:** angiomatous meningioma, brain anatomy, brain tumors cns tumors, general neurosurgery, neurosurgical procedures

## Abstract

Meningiomas are the most common intracranial benign tumors. Among them, cystic angiomatous meningiomas are very rare entities, making preoperative differentiation with other tumors challenging. We present a case of parasagittal angiomatous meningioma with a large associated cyst that mimicked a hemangioblastoma on preoperative imaging. It was treated surgically. We also performed a literature review and identified a total of 10 articles in the English language, describing a few similar cases of cystic angiomatous meningiomas. Conclusions: This report of a rare case of cystic angiomatous meningioma with a unique cystic presentation on preoperative imaging adds to the scarce body of literature on the topic. Surgery is ultimately the key method to make a diagnosis and to decompress the brain parenchyma in these patients.

## Introduction

Angiomatous meningiomas represent an uncommon subtype among meningiomas, which are the most common intracranial benign tumors [1]. Angiomatous meningiomas account for only about 2.1% of all cases [2-5], and their most distinctive characteristics are that the tumor is rich in vasculature [2] and often shows considerable peritumoral edema on preoperative MRI [5]. In rare cases, angiomatous meningiomas can have a cystic appearance [6,7], making preoperative differentiation with other tumors difficult. We report a case of a supratentorial extra-axial lesion, composed of a contrast-enhancing nodule adjacent to a large cyst, with no signs of cerebral edema. While the lesion was initially assumed to be a hemangioblastoma, histopathological assessment revealed an angiomatous meningioma.

Written informed consent was obtained from the patient for the publication of any potentially identifiable images or data.

## Case presentation

Case description

**Figure 1:**
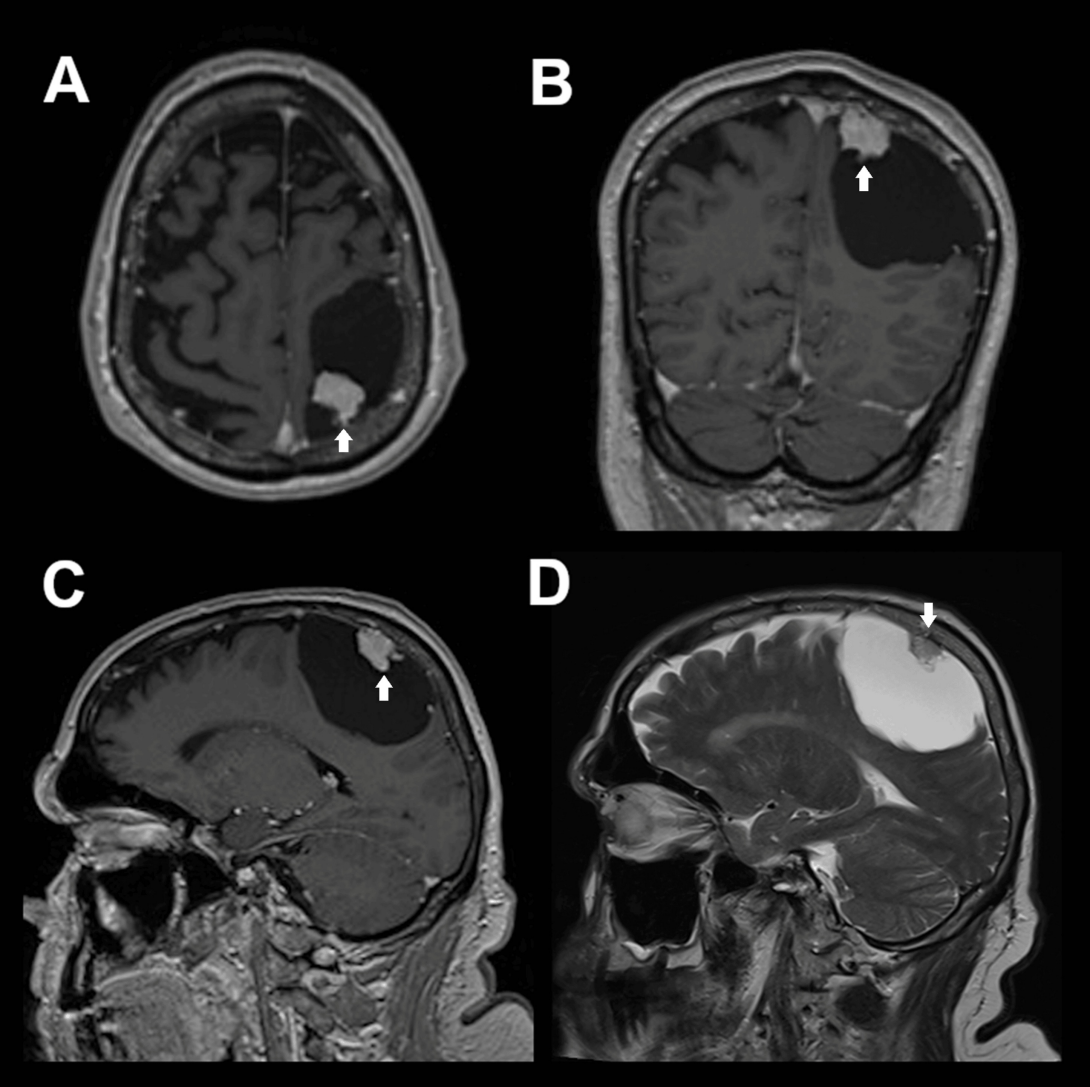
Preoperative MRI findings The images revealed a small contrast-enhancing lesion (white arrows) together with a large associated cyst in the paramedian left central region, suggestive of a hemangioblastoma (postcontrast T1 sequences in axial (A), coronal (B), and sagittal (C) views). T2 sequences did not show any signs of edema (D) MRI: magnetic resonance imaging

The patient was an 80-year-old male who experienced a bout of nausea, which resulted in a fall with a short loss of consciousness, followed by altered mental status. Subsequently, shortly after a coronavirus disease 2019 (COVID-19) infection, the patient noticed a deterioration of his handwriting, which led to a neurological work-up including a cranial MRI. The scan revealed a large intracranial extra-axial lesion in the left frontoparietal region, composed of a contrast-enhancing nodule with a paramedian localization at the level of the central sulcus and a large hypointense adjacent cyst that displaced the neighboring cerebral tissue, showing no signs of cerebral edema (Figure 1).

Following referral to our outpatient clinic, we initially suspected a supratentorial hemangioblastoma and recommended microsurgical resection. As for comorbidities, the patient suffered from arterial hypertension, type 2 diabetes, hypercholesterolemia, atherosclerosis of the left internal carotid artery, and osteoarthritis of both hips. Furthermore, he was on apixaban for paroxysmal atrial fibrillation, which was discontinued before surgery.

**Figure 2:**
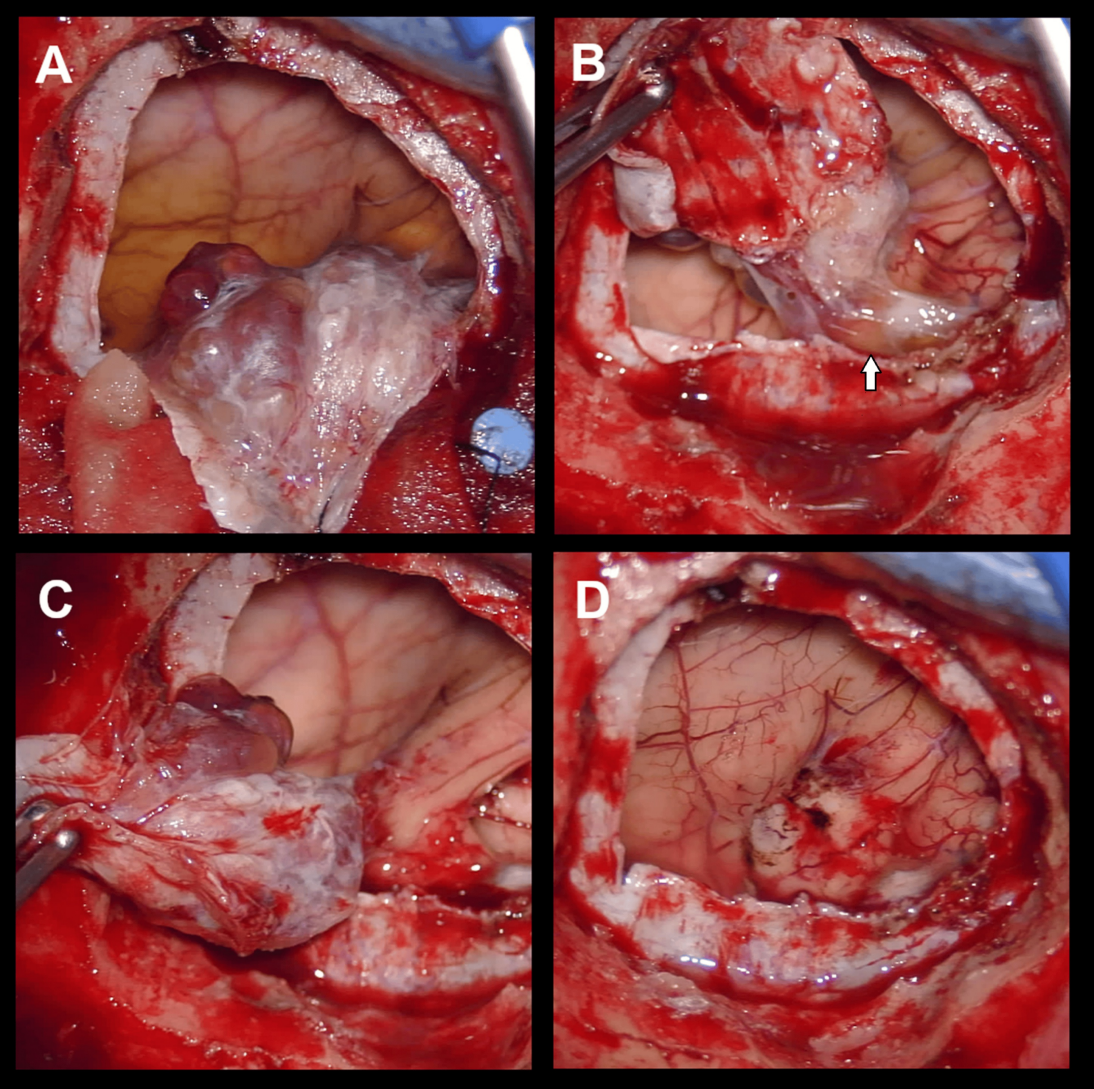
Intraoperative images after dural opening The images show the complete nodule with broad dural attachment (see white arrow in B). The xanthochromatous fluid of the cystic portion of the lesion can be observed (A). The lesion was removed together with the adhering dura. A small area of adhesion to the cerebral cortex was evident but without clear parenchymal invasion (B, C, and D)

Microsurgical resection was performed in a modified supine position. The patient was slightly turned to the right side, and a cushion was placed under his left shoulder and thorax, allowing for a 90-degree head turn and slight elevation. Neuronavigation was adopted to tailor the craniotomy just above the contrast-enhancing nodule. A curvilinear opening of the dura encompassed the entire nodule (Figure 2), which was completely resected together with the adhering dura. A small area of parenchymal adhesion was appreciated, but no signs of true invasive growth were detected intraoperatively. A xanthochromatous fluid was emptied from the cystic part, and during the inspection, no other suspicious tumor areas were identified. The dural defect was closed with a watertight duraplasty using galea.

The patient recovered well, and a postoperative CT scan ruled out any complications. In the later course, the patient described a short episode with rhythmic twitching of his right hand, which was interpreted as a focal seizure. He was subsequently started on levetiracetam. The rest of the postoperative course was uneventful.

Histopathological assessment

**Figure 3:**
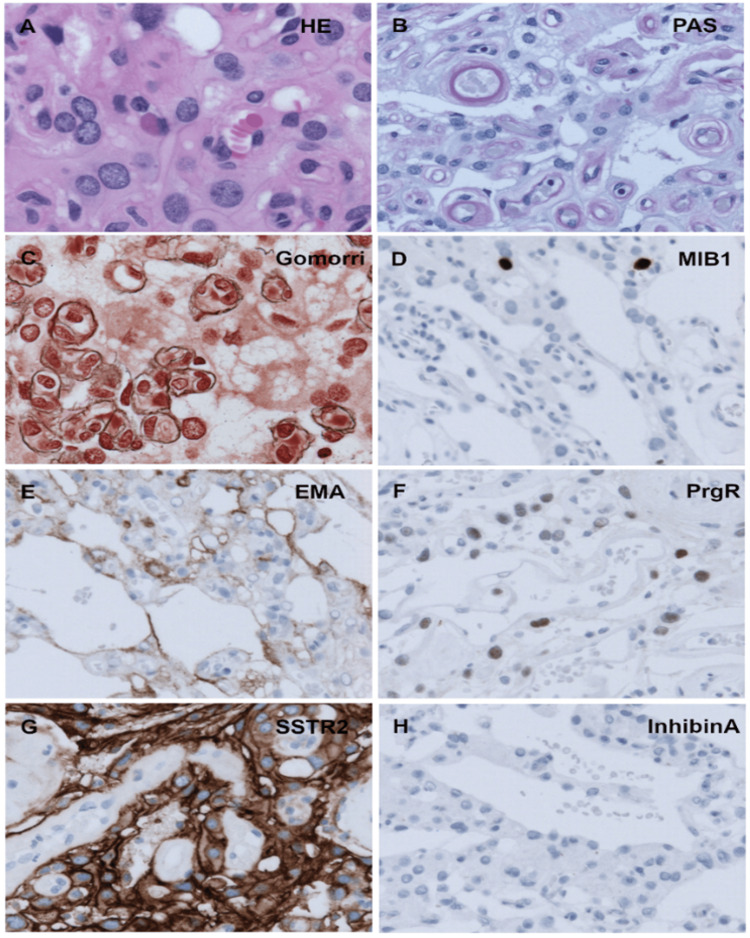
Histopathological examination Immunohistochemical features of a typical angiomatous meningioma with large meningothelial cells (HE) within many small capillaries (PAS) and absence of reticulin within the tumor cells (Gomori) were observed. Low MIB-1 proliferative activity and positivity of epithelial membrane antigen (EMA), somatostatin receptor (SSTR2A), and progesterone receptor (PgR) support the diagnosis, and negative InhibinA stains rule out hemangioblastoma (A-H)

To our surprise, the histopathological assessment revealed an angiomatous meningioma (CNS WHO Grade 1), and not a hemangioblastoma as preoperatively suspected. The well-vascularized, capillary-rich tumor displayed epithelial membrane antigen (EMA)-positive interspersed meningothelial cells with low mitotic activity. Additionally, the tumor was positive for somatostatin receptor 2A and progesterone receptor and negative for panCK and InhibinA (Figure 3). No signs of brain invasion were seen during histopathological workup.

## Discussion

A closer look at angiomatous cystic meningiomas

Meningioma is the most common intracranial benign tumor [1], arising from the arachnoid cap cells of the meninges. Among these, angiomatous meningiomas represent an uncommon subtype [2-4], whose most distinctive characteristic is their richness in vasculature [2] and the frequent presence of considerable peritumoral edema on the preoperative MRI [6]. Angiomatous meningiomas account for 2.1-2.59% of all meningiomas [3-5], and a cystic appearance is frequently reported [7,8]. Cystic meningiomas are a rare phenotypical variant of intracranial meningiomas, accounting for approximately 2-4% of all meningiomas [9,10]. We performed a literature review using three different medical databases (PubMed - Medline, Cochrane Library, and Embase). We used the keywords "angiomatous", "Angiomatous cystic", and "Angiomatous cystic meningiomas". The inclusion criteria were as follows: papers in English involving adult human participants (age >18 years); cases of angiomatous meningiomas with associated cyst. The exclusion criteria were as follows: incomplete data; papers not in the English language. The literature review elicited a total of 10 articles describing cystic tumors with a histological diagnosis of angiomatous meningioma, most of them supratentorial.

The age at presentation of angiomatous and cystic meningiomas varies, usually between the fourth to sixth decades of life [6-9], compared to the median age at diagnosis of 67 for meningiomas in general [1]. The age at diagnosis of cystic angiomatous meningiomas ranges from 42 years [1-27] to 80 years (our patient was 80 years old). Unlike cystic meningiomas in general, which typically exhibit a female predominance [8,9], the sex distribution in angiomatous meningiomas varies across case series [3-6]. The most commonly described sites of onset of angiomatous meningiomas are the brain convexity and parasagittal and falx regions, while it is less commonly seen at the skull base [3-6]. Similar locations are documented for cystic meningiomas [8,9]. Clinical presentation usually manifests progression due to the mass effect of adjacent brain parenchyma [3-8]. Angiomatous meningiomas seem to be associated with peritumoral parenchymal edema [4,5,6]. Hua et al. described different degrees of edema in their case series, which correlated with the vascular endothelial growth factor expression [6]. On the other hand, cystic meningiomas usually show a lesser degree of brain edema [9]. Interestingly, our case did not show peritumoral parenchymal edema.

Radiologically, angiomatous meningiomas are usually hypo/iso-intense in T1W sequences and hyperintense on T2W images, with a homogeneous post-contrast enhancement, except in the cases with cystic degeneration where contrast-enhancement can be heterogeneous (Table 1) [5,6,10].

**Table 1:** Clinical and radiological features of 11 documented patients with a brain cystic angiomatous meningioma NS: not specified

Study	Year	Number of patients	Patient sex	Age at diagnosis	Location	Symptoms	Enhancement	Dural tail sign	Peritumoral brain edema	Extent of excision
Liu et al. [3]	2013	4	NS	NS	NS	NS	Yes (heterogeneous)	NS	NS	NS
Hua et al. [6]	2017	48	NS	NS	NS	NS	NS	NS	NS	NS
Xia et al. [7]	2023	1	M	61	Supratentorial (left frontal)	Right limb weakness	Yes	NS	NS	NS
Boukobza et al. [8]	2016	1	NS	NS	NS	NS	NS	NS	Yes	NS
Go et al. [9]	2018	2	NS	NS	NS	NS	NS	NS	NS	NS
Wang et al. [12]	2016	1	M	57	Supratentorial (right frontal)	Left hemiparesis	Yes	NS	Yes	Total
Guan et al. [14]	2013	1	F	30	Infratentorial	Headache, vomiting	Yes	No	No	Total
Deb et al. [15]	2010	1	M	58	Infratentorial (cerebellopontine angle)	Vomiting, cerebellar symptoms	Yes	NA	Yes	Total
Pawan et al. [19]	2021	5	NS	NS	NS	Headache, seizures	NS	NS	Yes	NS
Bansal et al. [20]	2016	1	F	42	Supratentorial (right frontal)	Left hemiparesis	Yes	No	No	Total
Present case	2024	1	M	80	Supratentorial (left frontoparietal)	Deterioration of handwriting	Yes	No	Yes	Total

Cystic meningiomas are classified into four different morphologic types according to the Nauta classification [11]: type I: central cyst surrounded by macroscopic tumor; type II: peripheral cyst surrounded by a rim of microscopic tumor tissue; type III: peripheral cyst, outside the tumor and contained within the brain with a capsule of gliotic tissue, and type IV: cyst at the interface of brain parenchyma and tumor. According to the Nauta classification, our case was type IV, because no tumor was evident around the cyst. The exact mechanisms explaining cyst occurrence are still unclear. Intratumoral cysts could be secondary to cystic degeneration, ischemic necrosis, or hemorrhage, while peritumoral cysts may represent the result of trapped cerebrospinal fluid (CSF), an arachnoid cyst, or peripheral tumor degeneration. Cysts can also result from the secretion of fluid by the tumor cells [12].

Intracranial lesions mimicking hemangioblastoma

The preoperative differentiation between cystic meningiomas and other tumors can be difficult, and the final histopathological examination can lead to surprises [13]. The radiological features of a large cyst with contrast-enhancing mural nodule can mimic the appearance of a hemangioblastoma, as in our case, although the most frequent location of the latter is infratentorial [14-18]. Both angiomatous meningiomas and hemangioblastomas are classified as benign neoplasms (CNS WHO grade 1), although meningiomas are extra-axial lesions, while hemangioblastomas are intra-axial, which has implications for the surgical planning and technique. Typically, hemangioblastomas are located in the posterior fossa [18], while cystic angiomatous meningiomas are more often located supratentorially [19-24] (Table 1).

The histopathological diagnosis of hemangioblastoma may lead to a suspicion of Von Hippel-Lindau disease [16,18], which makes further diagnostic workup necessary. Other lesions also need to be differentiated from hemangioblastomas, with which they share cystic features, such as pilocytic astrocytomas [25], pleomorphic xanthoastrocytomas [26], and gangliogliomas [27]. Pilocytic astrocytomas are typically located in the cerebellum, and less commonly at the level of the optic chiasm, nerve or tract and the third ventricle; pleomorphic xanthoastrocytomas usually appear as cortical supratentorial tumors with a cystic component, with an intense contrast enhancement and sometimes with a positive dural tail sign; typically found in young patients. Gangliogliomas are common in young patients (aged less than 30 years); they are typically located in the temporal hemisphere and are usually associated with a history of temporal lobe epilepsy.

## Conclusions

Among meningiomas, angiomatous cystic types represent a unique entity, and they are rarely described in the literature; therefore, a differential diagnosis with other intracranial lesions is critical. Our report illustrated how a cystic angiomatous meningioma can mimic an intra-axial lesion like hemangioblastoma. This highlights the level of uncertainty when encountering a cystic intracranial tumor. Ultimately, surgery, as in our case, is the main method for obtaining a definitive histological diagnosis, as well as decompression of brain parenchyma in cases with large cysts.
